# Disease activity in paediatric vasculitis: development of a generic assessment tool - PVAS

**DOI:** 10.1186/1546-0096-9-S1-P92

**Published:** 2011-09-14

**Authors:** P Dolezalova, PA Brogan, S Özen, S Benseler, J Anton, J Brunner, DA Cabral, R Cimaz, KM OÂ´Neil, C Wallace, N Wilkinson, R Luqmani

**Affiliations:** 1 Charles University and General University Hospital in Prague, Czech Republic; 2 Institute of Child Health and Great Ormond Street Hospital, London, UK; 3 Hacettepe University, Ankara, Turkey; 4 The Hospital for Sick Children, University of Toronto, Canada; 5 Hospital Sant Joan de Déu, Universitat de Barcelona, Spain; 6 Innsbruck Medical University, Austria; 7 BC Children's Hospital and University of British Columbia, Canada; 8 AOU Meyer, Firenze, Italy; 9 University of Oklahoma Health Sci. Cyr., USA; 10 University of Washington and Seattle Children's Hospital, USA; 11 Nuffield Orthopaedic Centre, Oxford, UK; 12 NIHR Musculoskeletal Biomedical Research Unit, University of Oxford, UK

## Background

Primary systemic vasculitides (PSV) in childhood are rare but are associated with high morbidity/mortality. We adapted the adult Birmingham Vasculitis Activity Score (BVAS3), for use in childhood vasculitis: the Paediatric Vasculitis Activity Score (PVAS).

## Aim

To develop and validate PVAS based on modifications of BVAS3, and to establish an effective training system for use of PVAS according to the recommendations of the European Vasculitis Study Group (EUVAS).

## Methods

The PRINTO vasculitis classification registry was reviewed for the presence of items not included in the original nine organ systems in BVAS3; items that were present in ≥ 20% of patients were added. Critical review of these items by a working group of paediatric rheumatologists with an interest in vasculitis resulted in the first version of PVAS. During consensus meetings content and face validity was established, resulting in minor modifications to the PVAS and its glossary. The score weightings were unchanged from BVAS3 resulting in the same range of numeric scores: 0-63, where zero indicates no vasculitis disease activity. Twenty paediatric paper training cases were prepared for the initial tool assessment.

## Results

Eight paediatric-relevant items and their definitions were added to the original BVAS3 in the cutaneous (4), cardiovascular (3) and abdominal (1) systems. The final score for each organ system remained unchanged. Additionally, 22/56 BVAS3 items were re-defined for paediatric use, including definitions of weight loss and renal manifestations. Based on an ideal consensus answer for each of the 20 paper cases 9/11 investigators reached >85% agreement. The main cause of disagreement was the failure to record the absence of any items within a category.

## Conclusion

PVAS development marks an important international collaborative step towards a systematic and reliable clinical score of vasculitis disease activity for children. Ongoing work is assessing the utility of PVAS in real paediatric vasculitis patients, with the ultimate aim of using PVAS in routine clinical practice, and as a disease outcome measure in future clinical trials.

